# Two Rab5 Homologs Are Essential for the Development and Pathogenicity of the Rice Blast Fungus *Magnaporthe oryzae*

**DOI:** 10.3389/fpls.2017.00620

**Published:** 2017-05-05

**Authors:** Cheng D. Yang, Xie Dang, Hua W. Zheng, Xiao F. Chen, Xiao L. Lin, Dong M. Zhang, Yakubu S. Abubakar, Xin Chen, Guodong Lu, Zonghua Wang, Guangpu Li, Jie Zhou

**Affiliations:** ^1^Fujian Province Key Laboratory of Pathogenic Fungi and Mycotoxins and College of Life Sciences, Fujian Agriculture and Forestry UniversityFuzhou, China; ^2^State Key Laboratory of Ecological Pest Control for Fujian and Taiwan Crops, Fujian Agriculture and Forestry UniversityFuzhou, China; ^3^College of Ocean Science, Minjiang UniversityFuzhou, China; ^4^Department of Biochemistry and Molecular Biology, University of Oklahoma Health Sciences Center, Oklahoma CityOK, USA

**Keywords:** RabGTPases, MoRab5A, MoRab5B, endocytosis, pathogenesis, *Magnaporthe oryzae*

## Abstract

The rice blast fungus, *Magnaporthe oryzae*, infects many economically important cereal crops, particularly rice. It has emerged as an important model organism for studying the growth, development, and pathogenesis of filamentous fungi. RabGTPases are important molecular switches in regulation of intracellular membrane trafficking in all eukaryotes. MoRab5A and MoRab5B are Rab5 homologs in *M. oryzae*, but their functions in the fungal development and pathogenicity are unknown. In this study, we have employed a genetic approach and demonstrated that both MoRab5A and MoRab5B are crucial for vegetative growth and development, conidiogenesis, melanin synthesis, vacuole fusion, endocytosis, sexual reproduction, and plant pathogenesis in *M. oryzae*. Moreover, both MoRab5A and MoRab5B show similar localization in hyphae and conidia. To further investigate possible functional redundancy between MoRab5A and MoRab5B, we overexpressed *MoRAB5A* and *MoRAB5B*, respectively, in *MoRab5B:RNAi* and *MoRab5A:RNAi* strains, but neither could rescue each other’s defects caused by the RNAi. Taken together, we conclude that both MoRab5A and MoRab5B are necessary for the development and pathogenesis of the rice blast fungus, while they may function independently.

## Introduction

Rice blast is one of the most devastating diseases of rice in the world, directly threatening the global food security. The disease causing fungus, *Magnaporthe oryzae*, has become an important model organism for studying the mechanism of growth, development and pathogenesis of filamentous fungal pathogens and plant-microbe interactions (Valent and Chumley, 1991; Talbot, 2003). Multiple cellular functions, including intracellular membrane trafficking, are involved in the infection process of the fungus, which consists of multiple steps including conidial germination, appressorial differentiation, effector secretion, penetration peg formation and infectious growth (Song et al., 2010; Dou et al., 2011; Qi et al., 2016).

RabGTPases are key regulators of the membrane trafficking system, endocytosis and exocytosis in all eukaryotes (Hutagalung and Novick, 2011). All Rab proteins have five conserved GTP-binding motifs (G1–G5) and five conserved Rab family domains (F1–F5), which distinguish Rabs from other members of the RasGTPase superfamily (Pereira-Leal and Seabra, 2000; Bhuin and Roy, 2014). Rabs function as molecular switches by conformational changes between active GTP-bound and inactivate GDP-bound states, with only the GTP-bound active state capable of interacting with downstream effectors to promote diverse functions in membrane trafficking processes, such as vesicle formation, transport, docking, tethering, and fusion (Grosshans et al., 2006; Stenmark, 2009; Hutagalung and Novick, 2011). Recently, we reported that Rab proteins are involved in plant infection in *M. oryzae* and *Fusarium graminearum* (Liu et al., 2015; Zheng et al., 2015, 2016).

A total of 11 Rab/Ypts have been identified in the budding yeast *Saccharomyces cerevisiae*, while 66 exist in *Homo sapiens* and 57 in *Arabidopsis thaliana* (Lazar et al., 1997; Pereira-Leal and Seabra, 2001; Rutherford and Moore, 2002). Rab5 is the first well-documented member in the early endocytic pathway and promotes the fusion of early endosomes by interacting with its effectors *in vitro* (Gorvel et al., 1991) and *in vivo* (Bucci et al., 1992). Of the three Rab5 homologs Ypt51, Ypt52, and Ypt53 in the budding yeast, only *YPT51* is crucial for growth, endocytic traffic and vacuolar protein sorting, while *YPT52* or *YPT53* could partially restore the endocytosis defects caused by the deletion of *YPT51*, indicating functional redundancy to some extent (Singer-Krüger et al., 1994). In *Aspergillus nidulans*, RabA and RabB are orthologs of Ypt51 and Ypt52, respectively, and they also show same localization in early endosomes and partial overlapping functions. Both Δ*rabA* and Δ*rabB* impair growth of *A. nidulans*, however, unlike Δ*rabA*, Δ*rabB* can block early endosome movement and is the sole mediator of degradative endosomal identity. In addition, RabB and, to a lesser extent, RabA are capable of recruiting the CORVET complex to endosomal membranes (Abenza et al., 2009, 2010). Recently, two Rab5 homologs (FgRab51 and FgRab52) have been identified and characterized in *F. graminearum*, and their deletion mutants both show significant defects in hyphal growth, conidiation, and pathogenicity. Moreover, Δ*Fgrab51* almost completely recovers from growth deficiency when *FgRAB52* is introduced, while *FgRAB51* only partially rescues the defects caused by the deletion of *FgRAB52*, suggesting partial functional redundancy between *FgRAB51* and *FgRAB52* in *F. graminearum* (Zheng et al., 2015).

MoRab5A and MoRab5B of *M. oryzae* are orthologs of yeast Ypt51 and Ypt52, however, their functions in *M. oryzae* remain unknown. Our previous study has shown that the two Rab5 homologs show distinct biochemical properties (Qi et al., 2014), suggesting that MoRab5A and MoRab5B may play distinct biological roles in the fungus. In this study, we have characterized MoRab5A and MoRab5B through dominant negative (DN) and RNA interference (RNAi), and our results show that both Rabs are independently required for vegetative growth, conidiogenesis, and pathogenicity of *M. oryzae*.

## Materials and Methods

### Strains, Media, and Incubation Condition

The *M. oryzae* strains used in this study include Guy11, mating strain KA3, and relevant transformants based on Guy11 background. The yeast strains (wild-type BY4743 and single deletion mutant Δ*ypt51* based on diploid strain BY4743) were purchased from scientific community (EUROSCARF). Media used in the assays: complete media (CM) (0.6% yeast extract [w/v], 0.6% casein hydrolysate [w/v], and 1% sucrose [w/v]) and SYM (1% starch, 0.6% yeast extract, 0.3% sucrose, 2% agar) were for normal cultivation of *M. oryzae*. RBA (4% rice bran, 2% agar, pH 6.0∼6.5) was used for conidial production. OMA (5% oatmeal, 2% sucrose, 2% agar) was utilized in sexual cross assay. YPDA (2 g Tryptone, 1 g yeast extract, 20 μL 10 M NaOH, 2 g agar, in 95 mL distilled water, autoclaved, plus 5 mL filter-sterilized 40% glucose and 0.8 mL 100 × adenine) was used in yeast normal incubation. SD/-Ura [Clontech, 0.077 g DO Supplement-Ura, 3.7 g Minimal SD Base/Gal/Raf, 2 g agar, all in 100 mL of solution in double-distilled water (ddH_2_O) and autoclaved] was used in heterologous complementation. SPO^++^ (5 g yeast extract, 3 g Methyl acetate, plus ddH_2_O to 180 mL, autoclaved, plus 1.25 mL filter-sterilized 40% glucose and 20 mL 10 × amino acid) was applied in haploid mutant separation assay.

### Transformants of Point Mutation, Overexpression, RNAi, Heterologous Complementation, and GFP-Fusion

The *M. oryzae* protoplast preparation and fungal transformation were based on a previous description (Sweigard et al., 1998). The analysis of conservative domains and phylogenetic tree construction were performed by DNAMAN6.0 with amino acid sequences of Rab5 homologs in three different species. The cDNAs of *MoRab5A/B:DN* and *MoRab5A/B:OE* were obtained by PCR using specific primer pairs (Supplementary Table S1), then cloned into plasmid pTE11 with *Bam*HI/*Not*I to create *MoRab5A/B:DN* and *MoRab5A/B:OE* expression vectors. Special DNA sequences (synthesized in Bo Shang company, Hangzhou, China) analyzed by bio-application Beacon Designer 7.0 were used as templates to clone *MoRab5A/5BRNAi* sequence, then, respectively, linked into plasmid pSD1 with *Eco*RI/*Spe*I and *Xba*I/*Not*I to construct the *RNAi* vectors. The CDS of *MoRAB5A/B* were cloned with related primer pairs (Supplementary Table S1) and, respectively, linked into plasmid pYES2 with *Bam*HI/*Eco*RI to construct heterologous complementation vectors. MoRab5A/B native promoter, full CDS and GFP tag fragments were amplified using their respective relevant primer pairs (Supplementary Table S1) and fused together by splicing by overlap extension (SOE)-PCR. The GFP tag was fused to the N-terminus of MoRab5A or MoRab5B. Finally, the integrated fragments of GFP-MoRab5A/B were, respectively, inserted into pKNT plasmid with *Xho*I and *Eco*RI. All the sequences of expression vectors were sent to the company (Bo Shang, Hangzhou, China) to sequencing to ensure accuracy. The heterologous complementation vectors were transformed into Δ*ypt51* deletion mutant whereas other vectors were transformed into Guy11 strains. Moreover, we transformed the overexpression vectors of *MoRAB5A* and *MoRAB5B* into the *MoRab5B:RNAi* and *MoRab5A:RNAi* strains, respectively, to determine whether they could rescue the deficient function of each other. GFP-fusion vectors of MoRab5A/B were also transformed into Guy11 for analysis of their subcellular localization.

### Quantitative Real Time PCR

Strains involved in this experiment were inoculated in liquid CM and incubated at 28°C, 110 rpm for 3 days. Mycelia were then gathered from which total RNA was extracted and used to performed reverse transcription using the reverse transcription kit (Takara, 6210A) to generate cDNA to quantify expression levels with SYBR kit (Takara, DRR820A) using specific primers (Supplementary Table S1) with β-tubulin as endogenous reference gene. The data generated was finally analyzed with 2^-ΔΔC_T_^ method as previously reported (Livak and Schmittgen, 2001).

### Yeast Haploid Separation and Heterologous Complementation

The diploid strain (Δ*ypt51*) was cultured in SPO^++^ medium and shaked at 30°C, 180 rpm for 2–3 days until adequate single cells were observed under microscope. The cells were collected by centrifugation at room temperature, 7000 rpm for 1 min. Pellets were suspended and washed with distilled water, 0.5% β-glucuronidase was then added and incubated for 30 min to digest the cells for further separation. The suspension was transferred on YPDA plates and incubated at 30°C. After 2–3 days, single colonies were screened for Δ*ypt51* haploid strains by PCR using specific primers (Supplementary Table S1). The heterologous complementation vectors of *MoRAB5A/B* and empty pYES2 plasmid were transformed into haploid Δ*ypt51*, respectively. In addition, pYES2 was transformed into wild-type BY4743 to detect survival capacity of pYES2 on SD/-Ura plates. All the confirmed candidates screened using related primers (Supplementary Table S1) were determined on the SD/-Ura [containing 200 μg/mL CFW (Calcofluor White)] plates at 30°C for 3 days.

### Staining and Visualization by Microscopy

The related strains grown on SYM plates were placed on sterilized coverslips and incubated at 28°C. After 2 days, the hyphae on the coverslips were stained with CFW (35 μg/mL) for 5 min, washed with distilled water and observed under an Olympus Bx51 Microscope using two modes: bright field and UV excitation (300 nm). The hyphae were then stained with FM4-64 (8 μM) for 5 min, washed with distilled water and observed under the red excitation (561 nm). Aerial hyphae grown on RBA medium were stripped out after the plates were filled up with the hyphae. Mycelia blocks (2 cm × 0.5 cm × 0.25 cm) were cut and placed on microslides with the side bearing the hyphae directly resting on the slide surface and incubated under light for 1.5 days at 28°C. After this period, the slides were stained with lactophenol cotton blue solution (20 mL phenol, 0.6 g cotton blue, 44 mL glycerinum, 16 mL lactic acid, plus distilled water to 100 mL and diluted threefolds before using) for 5 min. Finally, the slides were washed with distilled water and visualized under Olympus Bx51 Microscope using bright field mode. For transmission electron microscopy manipulation, refer to previous description (Dou et al., 2011).

### Phenotypic Analysis

For growth rate (or sensitivity) assays, 3 mm diameter mycelia blocks were transferred onto the middle of normal CM plates (containing 200 μg/mL CFW) and incubated at 28°C. The colony was measured by crossing method after 10 days. For conidia quantification, the strains were cultured on RBA medium plates at 28°C until hyphae filled up with the plates, then aerial hyphae were stripped out. After 2 days of light incubation, conidia were collected and counted using hemacytometer. For appressorium-like structure (ALS) assay, a coverslip containing aerial hyphae was placed on the surface of hydrophobic Gelbond film (BMA, USA) with a drop of distilled water as interface, incubated at 28°C in a dark and humid environment. The germinated ALS were observed at different time points (24, 48, and 72 h) using a microscope. For sexual mating assay, respectively, prepared co-incubation pair of mating strain KA3 and each relevant strain in one OMA plate, incubated at 28°C until their hyphal edges attached. The plates were transferred into 20°C chamber with successive light for 25 days, observed the perithecia and ascospores with microscope.

### Pathogenicity Assay

The plant materials used for pathogenicity assays were susceptible rice (CO39) and barley (Golden Promise). Conidia were collected from the experimental strains which could generate conidia, and their concentrations adjusted to 10^5^ conidia/mL with 0.02% Tween. The conidial suspension was then sprayed evenly on the rice plant and incubated at a dark and humid environment for 24 h at 28°C, and then transferred into light condition. Lesion status was observed after 7 days. For the strains that could not produce conidia, fresh mycelia blocks were inoculated on susceptible rice and barley leaves (each was divided into two arrays: intact and wounded), kept dark and humid for 24 h at 28°C, then transferred into light environment and finally observed the lesion conditions after 7 days. The rice root infection assays were performed based on a previous method (Dufresne and Osbourn, 2001).

### Invasive Hyphae Assay

To observe the invasive hyphal growth, both excised rice and barley leaves were used. The inoculated rice leaves were decolored using a solution consisting of phenol, chloroform, and acetic acid (6:3:1, V/V/V). The leaves were further washed with absolute and 75% ethanol, and finally visualized by bright-field microscopy. For barley leaves, the lower epidermises were directly collected for microscopic observation. In addition, invasive hyphae were also directly visualized by their autofluorescence under UV excitation (Sun et al., 2006).

## Results

### MoRab5A and MoRab5B Can Recover the Resistance to CFW in *S. cerevisiae* Δ*ypt51*

**FIGURE 1:**
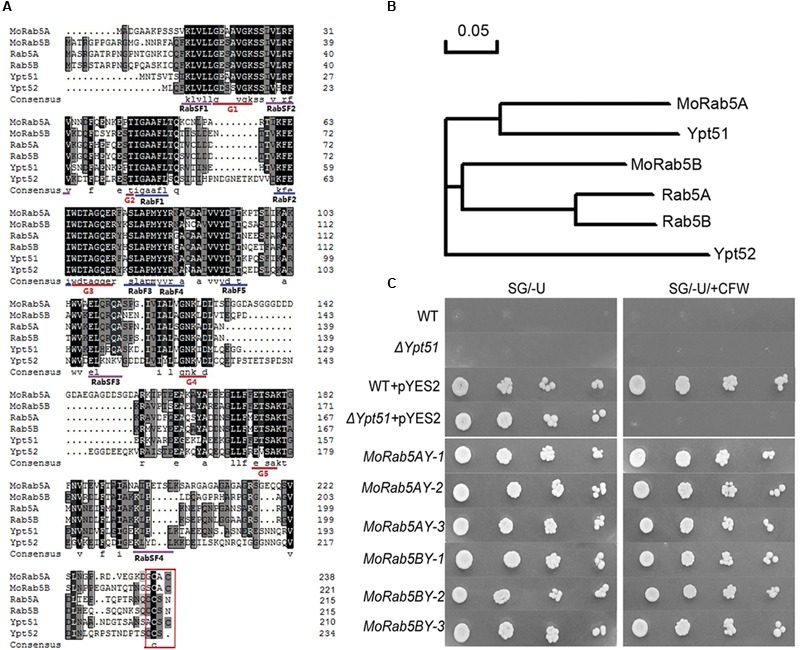
**Conserved domain analysis of MoRab5 and heterogeneous complementation in yeast. (A)** Multiple alignment of the amino acid sequences of Rab5 in *Magnaporthe oryzae* (MoRab5), Mammals (Rab5), and *Saccharomyces cerevisiae* (Ypt5). **(B)** Phylogenetic relationship analysis of Rab5 homologs in *M. oryzae* (MoRab5), Mammals (Rab5) and *S. cerevisiae* (Ypt5) by neighbor-joining method. **(C)** Heterogeneous complementation of MoRab5A and MoRab5B in yeast deletion mutant Δ*ypt51* with screening medium [SG/-U + 200 μg/mL Calcofluor White (CFW)]. The results were reproducible in three independent experiments. WT: wild-type BY4743, Δ*ypt51*: yeast deletion mutant *ypt51*, pYES2: expression plasmid, *MoRab5AY*: transformed *MoRAB5A* into Δ*ypt51, MoRab5BY*: transformed *MoRAB5B* into Δ*ypt51*.

We have identified two yeast Ypt51 (Vps21) homologs, MGG_06241 (vacuolar protein sorting-associated protein 21, NCBI accession XM_003717305.1) and MGG_01185 (GTP-binding protein Ypt5, NCBI accession XM_003714022.1) in *M. oryzae*, and named them as MoRab5A and MoRab5B, respectively (Qi et al., 2014). Protein sequence alignment showed that MoRab5A was much similar to Ypt51 with 57% identity (51% to Ypt52), while MoRab5B was more similar to Ypt52 with 57% identity (55% to Ypt51). To further understand the relationship of Rab5 homologs among *S. cerevisiae, M. oryzae*, and mammals, we identified five conserved GTP-binding motifs (G1 to G5), the Rab family motifs (RabF1 to RabF5) and the Rab subfamily motifs (RabSF1 to RabSF4) in MoRab5A and MoRab5B (**Figure 1A**), and conducted phylogenetic analysis among the Rab5 homologs (**Figure 1B**). The data suggested that MoRab5A and MoRab5B may have evolved to perform different functions.

To functionally characterize the relationship of Rab5 homologs between *M. oryzae* and *S. cerevisiae*, we investigated if *MoRAB5A* and *MoRAB5B* were able to rescue the defects of *ypt51* mutant in yeast. One of such defects was sensitivity to CFW which could bind to chitin and perturb the fungal cell wall (Lesage et al., 2005). In these complementation experiments, *MoRAB5A* and *MoRAB5B* were transformed, respectively, into the yeast deletion mutant Δ*ypt51*. Our results showed that both of the heterologous complementation transformants *MoRab5AY* and *MoRab5BY* survived on the SG/-U media containing 200 μg/mL CFW in contrast to the vector control (**Figure 1C**), indicating that both *MoRAB5A* and *MoRAB5B* can rescue the defect of Δ*ypt51* in response to CFW stress.

### MoRab5A and MoRab5B Are Crucial for Vegetative Growth

After many unsuccessful attempts to generate *MoRab5A* and *MoRab5B* deletion mutants by homologous recombination in *M. oryzae*, we constructed DN *MoRab5A*:N124I and *MoRab5B*:N133I as well as specific RNAi *MoRAB5A:RNAi* and *MoRAB5B:RNAi*. The resulting transformants were confirmed by DNA sequencing and further analyzed by qRT-PCR, showing about fourfold increase and 60% decrease, respectively, in target gene expression in the vegetative hyphae of the *DN* and *RNAi* strains, in comparison to Guy11, the wild-type strain (Supplementary Figure S1A). Importantly, the RNAi-mediated knockdown of either *MoRAB5A* or *MoRAB5B* was specific in the sense that *MoRab5A:RNAi* only reduced *MoRAB5A* expression without any effect on *MoRAB5B*, and *vice versa* (Supplementary Figure S1B). We have generated at least three independent strains for each *DN* or *RNAi* transformant, and their expression levels and phenotypes (see below) are similar in each case. Thus we have chosen one *DN* and one *RNAi* strain to show and represent the phenotypes of *MoRAB5A* and *MoRAB5B* deficiency, respectively, in our assays.

**FIGURE 2:**
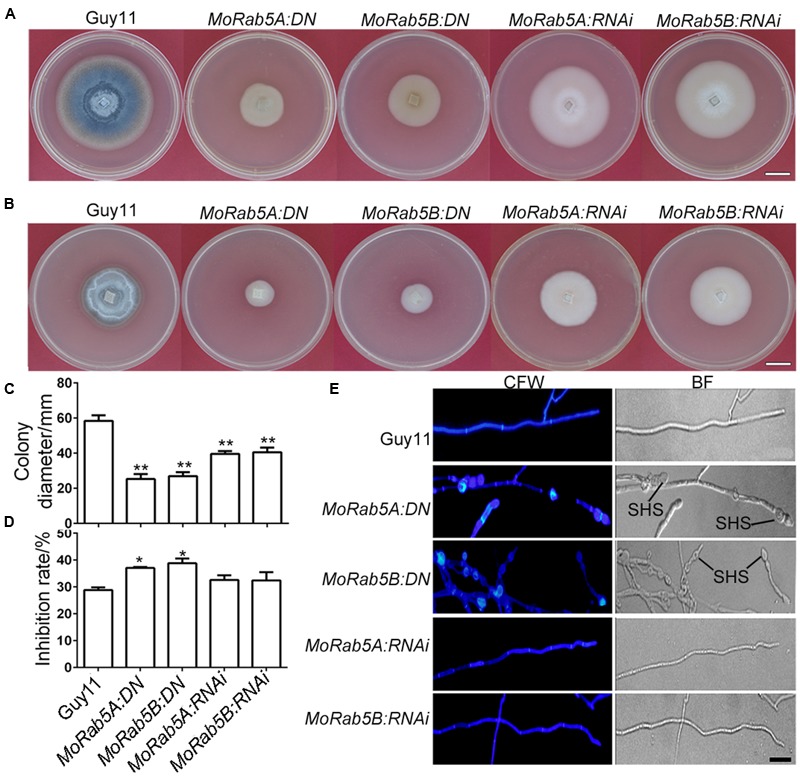
**MoRab5A and MoRab5B are involved in vegetative growth. (A)** The colony morphology of *MoRab5:DN* and *MoRab5:RNAi* strains grown on complete media (CM) for 10 days, scale bar = 2 cm. **(B)** The colony morphology of *MoRab5:DN* and *MoRab5:RNAi* strains grown on CM with 200 μg/mL CFW for 10 days, scale bar = 2 cm. **(C)** The colony average diameter of *MoRab5:DN* and *MoRab5:RNAi* strains on CM after 10 days. The significant difference was tested by two-way ANOVA with three repeats, ^∗∗^*p* < 0.01. **(D)** The inhibition rate of *MoRab5:DN* and *MoRab5:RNAi* strains grown on CFW-containing medium compared with Guy11. The colony diameter was measured after 10 days and the inhibition rate was counted using the following formula: (Colony diameter without CFW – Colony diameter with CFW)/Colony diameter without CFW), ^∗^*p* < 0.05. **(E)** Hyphae morphology of *MoRab5:DN* and *MoRab5:RNAi* strains. The 2-day hyphae grown on coverslips were stained with 35 μg/mL CFW for 5 min and washed with distilled water, then visualized under a microscope with the UV and BF modes. At least three independent strains for each DN or RNAi transformant were generated and characterized, and their phenotypes are essentially the same in each case. BF, bright field; SHS, swollen hyphae structure; scale bar = 20 μm.

We examined phenotypic changes in these *MoRab5A* and *MoRab5B* deficient strains and found that all the strains displayed declined hyphal growth, less aerial hyphae and disrupted melanin pigmentation on CM compared with Guy11 (**Figures 2A,C**), indicating that both MoRab5A and MoRab5B are essential for normal vegetative growth of *M. oryzae*. In *S. cerevisiae*, deletion of *YPT51* reduced cell resistance to CFW. To determine whether MoRab5A and MoRab5B are also important for cell wall integrity, both the *DN* and *RNAi* strains were grown on CM containing 200 μg/mL CFW, and their growth was all inhibited, especially the *MoRab5:DN* strains (**Figures 2B,D**). In addition, the *MoRab5A:DN* and *MoRab5B:DN* strains showed swollen aerial hyphae and obvious chitin deposition by 35 μg/mL CFW staining (**Figure 2E**), indicating abnormal chitin distribution pattern and consistent with the defects of *ypt51/52* deletion mutants in *S. cerevisiae*. Taken together, these data suggest that both MoRab5A and MoRab5B are crucial for vegetative growth of *M. oryzae*.

### MoRab5A and MoRab5B Are Involved in Endocytosis and Vacuole Fusion

**FIGURE 3:**
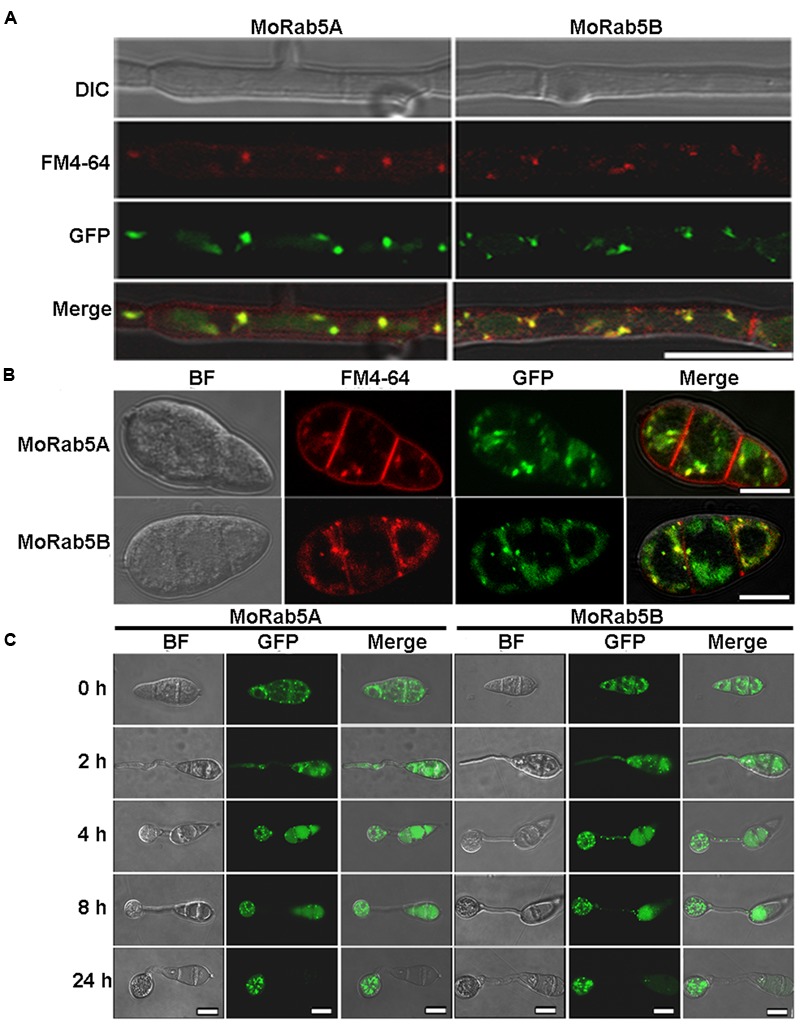
**Localization of MoRab5A and MoRab5B. (A,B)** The localization of MoRab5A and MoRab5B in hyphae and conidia. GFP-MoRab5A and GFP-MoRab5B were, respectively, co-localized with the endosome marker FM4-64 in punctate structures, scale bar = 10 μm. **(C)** The localization of MoRab5A and MoRab5B during conidial germination process at different germination time points. The results were reproducible in three experiments, scale bar = 10 μm.

Generally, Rab5 is a marker on the early endosomes (Chavrier et al., 1990; Abenza et al., 2009, 2010). We previously showed that MoRab5A and MoRab5B co-localized with human Rab5 on the early endosomes when expressed in mammalian cells (Qi et al., 2014). To examine the subcellular localization of MoRab5A and MoRab5B in *M. oryzae*, we constructed GFP-MoRab5A and GFP-MoRab5B fusion proteins under the control of their native promoters and transformed them into Guy11. GFP-MoRab5A and GFP-MoRab5B showed punctate pattern in mycelia, conidia, and developing appressoria (**Figures 3A–C**), consistent with their mammalian counterparts and suggesting that both MoRab5A and MoRab5B were localized to the early endosome in *M. oryzae*.

**FIGURE 4:**
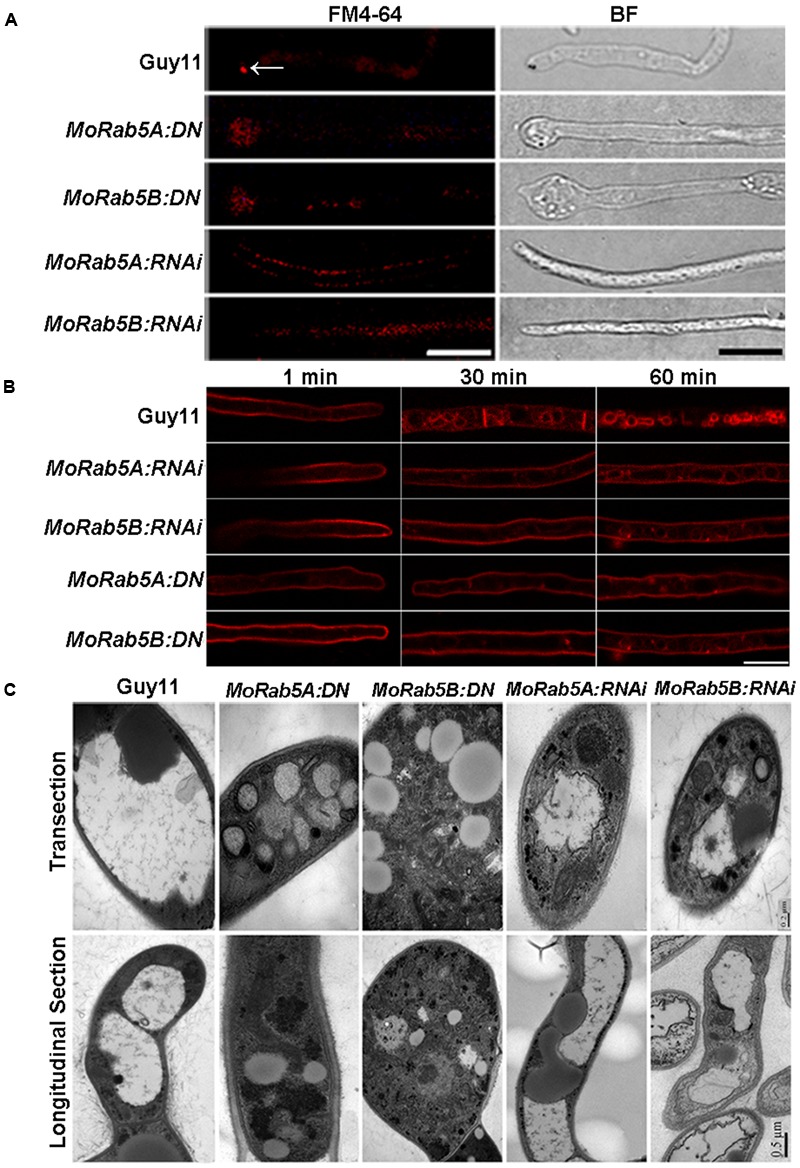
**MoRab5A and MoRab5B regulate endocytosis and vacuole fusion. (A)** *MoRab5:DN* and *MoRab5:RNAi* strains lost the endocytosis structure Spitzenkörper. Spitzenkörper stained with FM4-64 is indicated by white arrows, scale bar = 10 μm. **(B)** Internalization of FM4-64. Strains were incubated for 48 h in liquid CM, then stained with FM4-64 dye and observed under confocal microscopy after different time points, scale bar = 10 μm. **(C)** Smaller and more vacuoles observed in *MoRab5:DN* and *MoRab5:RNAi* strains. The vacuole visualization assay was performed with TEM (transmission electron microscope), scale bar = 0.2 μm (transection) or 0.5 μm (longitudinal section). The results were reproducible in three experiments.

In filamentous fungi, the Spitzenkörper is a special structure related to endocytosis at the apical region of hyphae (Grove and Bracker, 1970). To investigate whether MoRab5 is involved in endocytosis, we examined the formation of the Spitzenkörper at the hyphal tips of Guy11 and *MoRab5* deficient strains by monitoring FM4-64 endocytosis and labeling of Spitzenkörper. Results showed that the Spitzenkörper was readily detected in Guy11 but not in the *MoRab5* deficient strains (**Figure 4A**). Moreover, GFP-MoRab5A and GFP-MoRab5B-positive punctate structures co-localized with the endocytic marker FM4-64 (**Figures 3A,B**), consistent with the result that MoRab5 stimulated the internalization of fluid phase endocytic marker horseradish peroxidase (HRP) into the early endosomes in mammalian cells (Qi et al., 2014). In addition, FM4-64 was found on the plasma membrane after 1 min staining, and internalized into the endomembrane of hyphal cells of Guy11 within 30 min. By contrast, FM4-64 largely remained on the plasma membrane of all *MoRab5* deficient strains after 30 min, with only a small fraction of the endomembrane labeled with FM4-64 even after 1 h (**Figure 4B**). These results collectively suggested that MoRab5A and MoRab5B are involved in the endocytic pathway.

Rab proteins are involved in the fusion of vesicles and vacuole during vacuolar biogenesis in fungi (Hutagalung and Novick, 2011). In the budding yeast, a small but significant number of cells displayed fragmented vacuolar structures in *ypt5* deletion mutants (Singer-Krüger et al., 1994). To understand the function of MoRab5 in vacuolar biogenesis in *M. oryzae*, we examined vacuolar morphology in hyphae of the *MoRab5* deficient strains by transmission electron microscopy. The hyphae of the wild-type Guy11 were larger, and usually contained 1–2 large vacuoles. In contrast, the large vacuoles were absent in *MoRab5A:DN* and *MoRab5B:DN* strains, and instead there were smaller vesicles (**Figure 4C**), suggesting defective vesicle fusion and fragmentation of vacuoles in the cell.

### MoRab5A and MoRab5B Are Essential to Asexual Reproduction and Affect Sexual Reproduction

**FIGURE 5:**
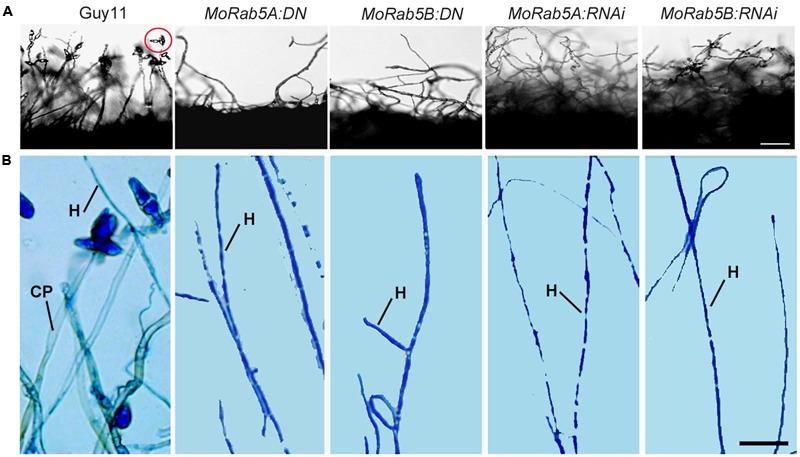
**MoRab5A and MoRab5B modulate conidiophore differentiation to control conidiation. (A)** Absence of conidia is observed in *MoRab5:DN* and *MoRab5:RNAi* strains. The conidia were visualized after 48 h after stripping out the aerial hyphae on RBA medium with light cultivation, scale bar = 50 μm. **(B)** Conidiophore differentiation was blocked in *MoRab5:DN* and *MoRab5:RNAi* strains. The hyphae grown on coverslips were stained with lactophenol cotton blue for 5 min, observed directly under microscope with bright field after water washing. The results were reproducible in three experiments. CP: conidiophore, stained to gray. H: hyphae, stained to blue. Scale bar = 20 μm.

In order to investigate the function of MoRab5 in conidiation, Guy11 and all *MoRab5* deficient strains were cultured on rice bran and oatmeal media for conidiation assay. Remarkably, no conidia were found in *MoRab5* deficient strains by microscopy, in contrast to the large number of conidia produced by Guy11 (**Figure 5A**). Then we used lactophenol cotton blue to stain conidiophore (gray-white) and mycelia (blue) (Zhou et al., 2009), and found that both conidiophore and mycelia were readily identified in Guy11, but only mycelia were present in all *MoRab5* deficient strains (**Figure 5B**), suggesting an important role for MoRab5A and MoRab5B in conidiophore differentiation.

**FIGURE 6:**
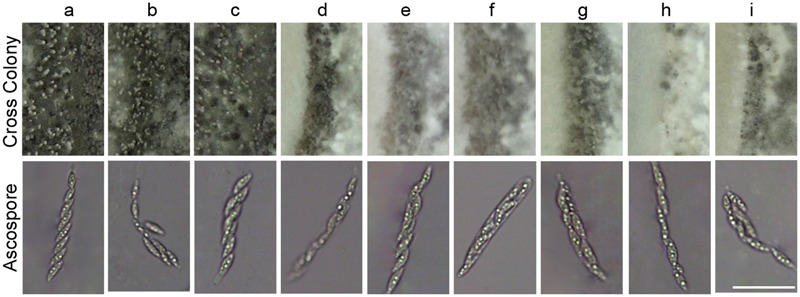
**MoRab5A and MoRab5B are involved in the sexual reproduction of *M. oryzae*.** The sexual mating assay. The *MoRab5* transformants and KA3 were co-cultured on OMA medium for 25 days, then photographed to analyze perithecia and ascospores. The results were reproducible in three experiments. Scale bar = 50 μm. a, *Guy11*; b, *MoRab5A:OE-3*; c, *MoRab5B:OE-27*; d, *MoRab5A:RNAi-4*; e, *MoRab5A:RNAi-MoRab5B:OE-1*; f, *MoRab5A:RNAi-MoRab5B:OE-52*; g, *MoRab5B:RNAi-5*; h, *MoRab5B:RNAi-MoRab5A:OE-11*; i, *MoRab5B:RNAi-MoRab5A:OE-20*.

In fission yeast *Schizosaccharomyces pombe*, the deletion of *YPT5* significantly reduced sexual mating efficiency (Tsukamoto et al., 2013). Hence, we conducted cross assay on oatmeal media to determine whether MoRab5A and MoRab5B are involved in sexual mating process in *M. oryzae*. After cultivating approximately 25 days at 20°C, a large number of perithecia were formed when Guy11 was crossed with KA3 strain, and eight ascospores were typically found in the asci by microscopy. Even though all the *MoRab5* deficient strains could still generate perithecia and ascospores, the number of perithecia were significantly decreased in comparison to Guy11 (**Figure 6**), suggesting that both MoRab5A and MoRab5B are also involved in sexual reproduction of *M. oryzae*.

### MoRab5A and MoRab5B Are Essential for Plant Infection by *M. oryzae*

**FIGURE 7:**
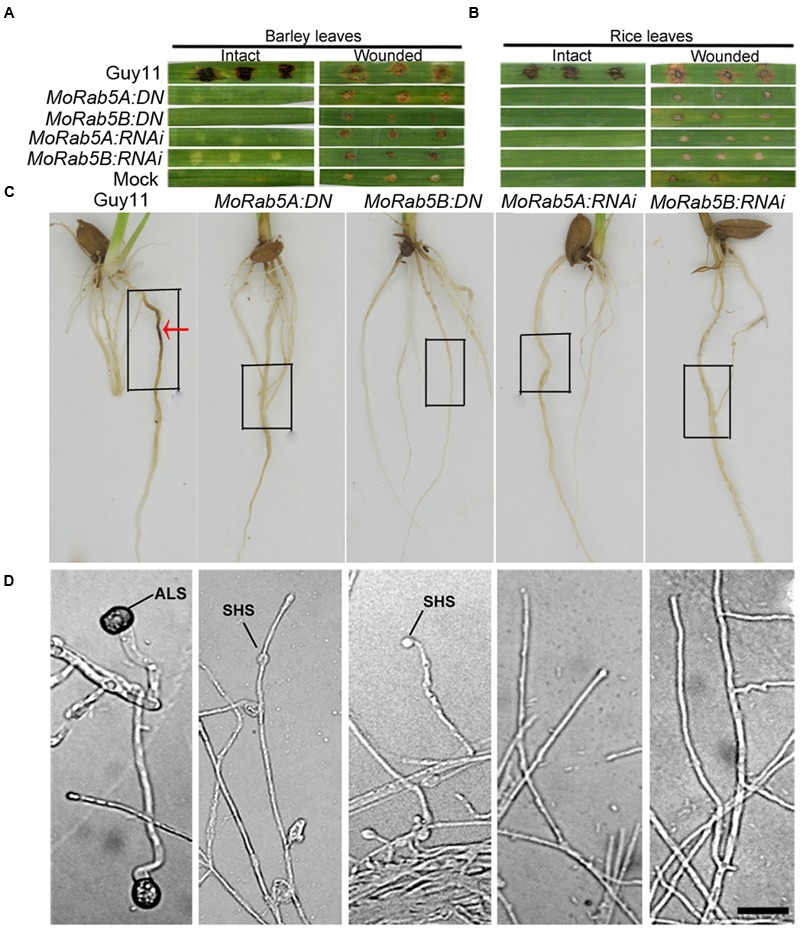
**MoRab5A and MoRab5B are essential for pathogenicity of *M. oryzae*. (A–C)** The pathogenicity was lost in *MoRab5:DN* and *MoRab5:RNAi* strains. The inoculation condition was visualized 7 days later for intact and wounded barley leaves **(A)** and intact and wounded rice leaves **(B)** and living rice roots **(C)**, mycelia blocks were covered in the black frame region. **(D)** The appressorium-like structure was blocked in *MoRab5:DN* and *MoRab5:RNAi* strains. The hyphae grown on coverslips were induced on hydrophobic film for 24, 48, and 72 h. The results were reproducible in three experiments. ALS, appressorium-like structure; SHS, swollen hyphae structure. Scale bar = 20 μm.

Appressorium development from conidia is an essential stage for the rice blast fungus to infect its hosts. However, none of the *MoRab5:DN* and *MoRab5:RNAi* strains could produce any conidia, we used mycelia plugs to inoculate intact and wounded barley leaves, rice leaves and roots, respectively. At 7 days post-inoculation (dpi) and 14 dpi, typical lesions (expanded and brown) were observed on rice and barley leaves inoculated with Guy11. In contrast, no disease lesions appeared on intact rice and barley leaves inoculated with *MoRab5:DN* and *MoRab5:RNAi* strains (**Figures 7A,B**), the results were the same on rice roots (**Figure 7C**). On the other hand, some small and brown scabs, distinct from typical lesions, were present on wounded barley and rice leaves (**Figures 7A,B**).

In order to determine if these brown scabs were confined disease lesions caused by invasive hyphae, tissues around the scabs (including the scabs) in rice leaves were cleaned and the lower epidermis of barley leaves were collected to observe through an Olympus Bx51 microscope. No bulbous invasive hyphae were found in the scabs from the leaves inoculated by the *MoRab5* deficient strains while bulbous invasive hyphae were abundant in the lesions from the leaves inoculated by Guy11 (Supplementary Figure S2A). It was reported that callose can emit detectable blue-green fluorescence under UV excitation and bulbous invasive hyphae are enriched with callose (Sun et al., 2006). To observe whether there is callose deposits in lesions, we directly examined the brown scabs by UV microscopy, no any blue-green fluorescence was detected in the scabs produced by the *MoRab5* deficient strains whereas strong blue-green fluorescence surrounding the bulbous invasive hyphae were observed in the disease lesions caused by Guy11 (Supplementary Figures S2B,C). Therefore, we concluded that full function of MoRab5A and MoRab5B is essential for the plant infection of *M. oryzae*.

Besides the appressoria developed from conidia, a special structure formed at the tips of hyphae on hydrophobic or plant surfaces, called ALS, can also trigger hyphal infection of susceptible hosts (Sun et al., 2006; Liu et al., 2010; Kong et al., 2013). The hyphae of *MoRab5* deficient strains were inoculated on the surface of hydrophobic film (Gelbond film from BMA company, USA), and no ALS emerged in any of the *MoRab5:DN* and *MoRab5:RNAi* strains even after 72 hpi, on the contrast, typical ALSs were readily detected at the tips of hyphae in Guy11 after 24 hpi (**Figure 7D**). Our results suggested that MoRab5A and MoRab5B are essential for the development of ALS, which may partially account for the pathogenicity defect in the *MoRab5* deficient strains.

### MoRab5A and MoRab5B Are Not Functionally Redundant

**FIGURE 8:**
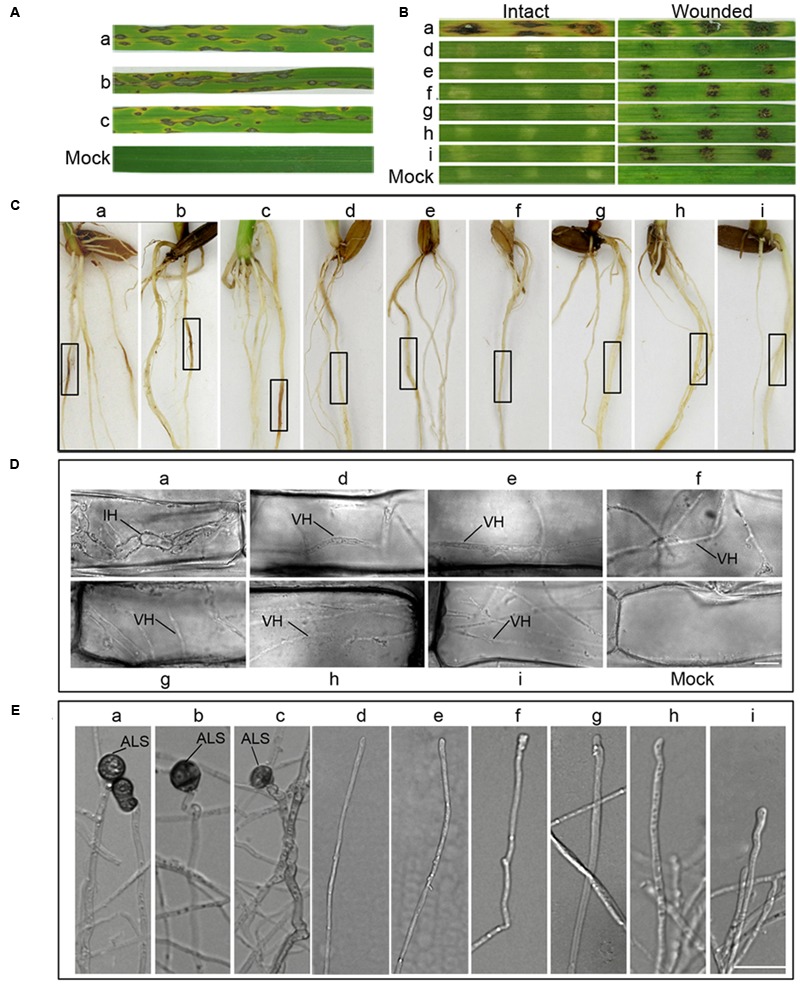
**Overexpression of *MoRAB5A* or *MoRAB5B* cannot recover the pathogenicity of *MoRab5B* or *MoRab5ARNAi* strains. (A)** Spray inoculation assay of *MoRab5:OE* in rice. Prepared conidia suspension (1 × 10^5^ conidia/mL) with 0.02% Tween, evenly sprayed on rice leaves, observing lesions after 7 days. **(B)** Barley leaves inoculation assay. Fresh mycelia blocks were covered on barley leaves and lesions observed 7 days later. **(C)** Rice root inoculation assay. The manipulation was displayed in material and procedures. Black frame indicates the inoculation region. **(D)** The visualization of invasive hyphae in wounded barley leaves. IH, invasive hyphae; VH, vegetative hyphae. Scale Bar = 10 μm. **(E)** The appressorium-like structure induction assay. The hyphae grown on coverslips were induced on hydrophobic film for 24, 48, and 72 h. The results were reproducible in three experiments. ALS, appressorium-like structure; SHS, swollen hyphae structure. Scale bar = 20 μm. a, Guy11; b, *MoRab5A:OE-3*; c, *MoRab5B:OE-27*; d, *MoRab5A:RNAi-4*; e, *MoRab5A:RNAi-MoRab5B:OE-1*; f, *MoRab5A:RNAi-MoRab5B:OE-52*; g, *MoRab5B:RNAi-5*; h, *MoRab5B:RNAi-MoRab5A:OE-11*; i, *MoRab5B:RNAi-MoRab5A:OE-20*.

There are three yeast Rab5 homologs, Ypt51, Ypt52, and Ypt53, and they have overlapping functions in *S. cerevisiae* (Singer-Krüger et al., 1994). MoRab5A and MoRab5B also exhibited similar functions based on our phenotypic analysis of vegetative growth, conidiogenesis, endocytosis, and pathogenicity. To test possible functional redundancy of the two Rab5 homologs in *M. oryzae*, we investigated if introduction of *MoRAB5A* into *MoRab5B:RNAi* strains can rescue the defects, and *vice versa*. To this end, we made overexpression constructs for *MoRAB5A* and *MoRAB5B* and transformed them into the protoplasts of the *MoRab5B:RNAi* and *MoRab5A:RNAi* strains, respectively, as well as Guy11. Positive transformants showed 5- to 15-fold increase the transcription of *MoRAB5A* and *MoRAB5B*, respectively, in the vegetative hyphae compared with Guy11 and parental RNAi strains by qRT-PCR analysis (Supplementary Figures S3A–C), although we could not distinguish between single and multiple copy insertion at this stage. Both *MoRab5A:OE* (overexpression of *MoRAB5A* in Guy11) and *MoRab5B:OE* (overexpression of *MoRAB5B* in Guy11) showed no effect on growth (Supplementary Figures S4A,B), conidiophore differentiation, conidiation (Supplementary Figures S5A–C), ALS formation (**Figure 8E**), Spitzenkörper development (Supplementary Figure S6), and sexual reproduction (**Figure 6**).

However, the *MoRab5* overexpression transformants in RNAi background, including *MoRab5A:RNAi-MoRab5B:OE* and *MoRab5B:RNAi-MoRab5A:OE* transformants produced small amounts of aerial hyphae and colorless colonies, which were essentially the same phenotypes as the *RNAi* strains, suggesting that *MoRAB5A* and *MoRAB5B* cannot recover each other’s functions in growth and pigmentation (Supplementary Figures S4A,B). Moreover, overexpression of *MoRAB5A* or *MoRAB5B* was unable to recover each other’s functions in conidial production, conidiophore differentiation (Supplementary Figures S5A–C), ALS formation (**Figure 8E**), Spitzenkörper development (Supplementary Figure S6), and sexual reproduction (**Figure 6**).

Since *MoRab5A:OE* and *MoRab5B:OE* in wild-type background could produce conidia normally, we inoculated live leaves of CO39 cultivar by conidia (10^5^/mL) spraying, typical lesions were observed on the leaves at 7 dpi (**Figure 8A**). Similar results were obtained when using mycelia plugs to inoculate the roots of CO39 cultivar (**Figure 8C**), suggesting that overexpression of *MoRAB5A* or *MoRAB5B* has no negative effect on the pathogenicity of *M. oryzae*. To determine if overexpression of *MoRAB5A* or *MoRAB5B* can rescue the pathogenicity of the *RNAi* strains, mycelia of *MoRab5A:RNAi-MoRab5B:OE* and *MoRab5B:RNAi-MoRab5A:OE* tranformants were used to inoculate intact and wounded barley leaves, and there were still no expanded disease lesions at 7 dpi. However, there were brown scabs in the wounded leaves (**Figure 8B**), which were different from the brown scabs produced by the parental *RNAi* strains (**Figures 7A,B**). Through microscopic examination, we found that these scabs were simply vegetative hyphae retained in the wounded leaf surface, rather than typical swollen invasive hyphae in the leaves inoculated by Guy11 (**Figure 8D**). Taken together, these results suggested that MoRab5A and MoRab5B could not complement each other in regulating the development and pathogenesis of *M. oryzae*.

## Discussion

In this study, we found that both MoRab5A and MoRab5B are localized in punctate structures, similar to their homologs in mammalian, yeast, and other fungal cells (Bucci et al., 1992; Armstrong et al., 1993; Singer-Krüger et al., 1994; Zheng et al., 2015), and consistent with their co-localization with Rab5 in early endosomes when expressed in mammalian cells (Qi et al., 2014). Moreover, we have functionally characterized *MoRAB5A* and *MoRAB5B* through point mutation and RNAi-mediated knockdown in *M. oryzae*, and shown that both are required for vegetative growth, conidiogenesis, vacuolar fusion, and pathogenicity to rice and barley.

The Spitzenkörper is a specialized structure formed by apical growth of hyphae, and it is related to endocytosis (Grove and Bracker, 1970). FgRab51 and FgRab52 regulate endocytosis in *F. graminearum*, and deletion of either gene can block the formation of Spitzenkörper and internalization of plasma membrane protein UapC (Zheng et al., 2015). Disruption of *MoRAB5A* and *MoRAB5B* by DN or RNAi leads to similar defects and impairs the internalization of endocytic marker FM4-64. Moreover, Qi et al. (2014) have shown that overexpression of either *MoRAB5A* or *MoRAB5B* can stimulate the endocytosis of HRP. Our data suggest that MoRab5A and MoRab5B are indeed involved in the endocytic pathway in *M. oryzae*. Endocytosis is crucial for the uptake and signal transduction of extracellular substances and plasma membrane-associated proteins (Sorkin and von Zastrow, 2009; Goode et al., 2015) and is evolutionally conserved. Deficient endocytosis has been shown to block fungal hyphal growth due to failure of recycling cellular components, such as cell wall-building enzymes, to the apical membrane (Upadhyay and Shaw, 2008; Higuchi et al., 2009; Song et al., 2010). Our data show that *MoRab5A*/*B:DN* strains reduce hyphal growth and accumulate CFW in swollen hyphae. This observation suggested that endocytic deficiency may abrogate the endocytosis of some extracellular materials into the cell or block the recycling of cell wall components, such as chitin synthases, to the hyphal apex, leading to their accumulation in the swollen hyphae of the fungus.

In the fungal pathogen *Ustilago maydis*, the endocytosis-associated gene *YUP1* is localized to the early endosome and is important for the early stage of pathogenic development (Fuchs et al., 2006). In *F. graminearum*, deletion of endocytosis-related genes *FgRAB51* and *FgRAB52* results in the complete lost of the pathogenicity to wheat (Zheng et al., 2015). The *M. oryzae* SNARE protein MoSyn8 participates in endocytosis, its deletion causes defects in infection of the host plant (Qi et al., 2016). In our study, *MoRab5A* and *MoRab5B DN* and *RNAi* strains inhibit the endocytosis of FM4-64, the formation of Spitzenkörper and the infection of rice and barley, suggesting that the disruption of endocytosis also lead to defect in pathogenicity of *M. oryzae*. Although the relationship between endocytosis and metabolism has not been characterized in *M. oryzae*, we speculate that the disruption of *MoRAB5A* and *MoRAB5B* may block the endocytosis of a number of receptors and transporters on the cell surface and change the metabolic activity such as glucose homeostasis, resulting in reduced hyphal growth and appressorium development in response to cues from the host.

The vacuole of a fungal cell is a complex organelle with various functions include maintaining cytosolic ion homeostasis and carrying out the degradation of lipid reserves, and may produce osmotically active metabolites, such as glycerol that can generate enormous turgor pressure critical for penetrating plant cuticle. In rice blast fungus, several proteins involved in vacuole fusion have been identified. Disruption of the vacuolar SNARE proteins MoSec22 and MoVam7 results in decreased conidiation, endocytosis, and pathogenicity (Song et al., 2010; Dou et al., 2011). In addition, MoMon1 and the small GTPase MoYpt7 are also essential for vacuolar fusion, conidiation, and virulence (Gao et al., 2013; Liu et al., 2015). In this study, *MoRAB5A* and *MoRAB5B DN* strains cause small fragmented vacuoles, suggesting that *MoRAB5A* and *MoRAB5B* are essential for vacuole biogenesis in *M. oryzae*.

In *S. cerevisiae*, Ypt51p/Vps21p, Ypt52p, and Ypt53p show overlapping functions, but Ypt51p/Vps21p plays a major role in all analyzed phenotypes (Horazdovsky et al., 1994; Singer-Krüger et al., 1994). Even though MoRab5A and MoRab5B are highly homologous to Ypt51/Vps21 and Ypt52, respectively, both of them can rescue the defects of Δ*ypt51* deletion mutant. Their *DN* and *RNAi* strains show similar defects in all analyzed phenotypes, but the defects of *MoRab5A:RNAi* cannot be rescued by overexpression of *MoRAB5B*, and *vice versa*, indicating that MoRab5A and MoRab5B are not functionally redundant in *M. oryzae*. These results are different from their homologs in *A. nidulans* and *F. graminearum* (Abenza et al., 2010; Zheng et al., 2015). Furthermore, we previously found that MoRab5A and MoRab5B show similar endocytosis function when expressed in mammalian cells. Both of them co-localize with human Rab5 to early endosomes, but MoRab5B is different from MoRab5A in the sense that it can promote early endosome fusion, display higher intrinsic GTPase activity and stimulate fluid phase endocytosis (Qi et al., 2014). Like hRab5, both MoRab5B and FgRab52 contain a Ser residue in the switch I region through amino acid sequence alignment, and they share similar functions with Rab5, suggesting that they are more homologous to hRab5. In contrast, Ypt51, MoRab5A, and FgRab51 contain a Pro residue at the corresponding position, and they are more similar to another human Rab protein Rab22, which regulates early endosomal sorting and recycling (Qi et al., 2014; Zheng et al., 2015). Taken together, these results suggest that MoRab5A and MoRab5B belong to the Rab5 subfamily, but they are not fully functionally redundant.

## Author Contributions

JZ and GPL conceived and designed the experiments. CY, XD, HZ, XFC, XL, and XC performed the experiments. CY and XD wrote the manuscript. JZ, GPL, ZW, GDL, YA, DZ, and XFC revised and approved the manuscript.

## Conflict of Interest Statement

The authors declare that the research was conducted in the absence of any commercial or financial relationships that could be construed as a potential conflict of interest.
