# Supplementary material for: Two Rab5 Homologs Are Essential for the Development and Pathogenicity of the Rice Blast Fungus Magnaporthe oryzae
Source: Front Plant Sci. 2017 May 5;8:620. doi: 10.3389/fpls.2017.00620 (PMC5418346; doi:10.3389/fpls.2017.00620)
Supplement: Supplementary file 1 [file Data_Sheet_1.doc]

**Two Rab5 homologs are essential for the development and pathogenicity of the rice blast fungus *Magnaporthe oryzae***

Chengdong Yang^1,2^¶, Xie Dang^1^¶, Huawei Zheng^1,2^, Xiaofeng Chen^2^, Xiaolian Lin^1,2^, Dongmei Zhang^2^, Yakubu Saddeeq Abubakar^1,2^, Xin Chen^1^, Guodong Lu^2^, Zonghua Wang^1,2,3^, Guangpu Li^4＊^ and Jie Zhou^1,2＊^

1 Fujian Province Key Laboratory of Pathogenic Fungi and Mycotoxins and College of Life Sciences, Fujian Agriculture and Forestry University, Fuzhou, 350002, China.

2 State Key Laboratory of Ecological Pest Control for Fujian and Taiwan Crops, Fujian Agriculture and Forestry University, Fuzhou, 350002, China.

3 College of Ocean Science, Minjiang University, Fuzhou, 350108, China.

4 Department of Biochemistry and Molecular Biology, University of Oklahoma Health Sciences Center, Oklahoma City, Oklahoma, United States of America.

¶These authors contributed equally to this work

＊Authors for Correspondence: Drs. Jie Zhou, Guangpu Li

E-mail: jiezhou@fafu.edu.cn, Guangpu-Li@ouhsc.edu

Running title: Rab5s in *Magnaporthe oryzae*

Keywords: Rab GTPases, MoRab5A, MoRab5B, endocytosis, pathogenesis, *Magnaporthe oryzae*

**SUPPLEMENTARY MATERIAL**

**
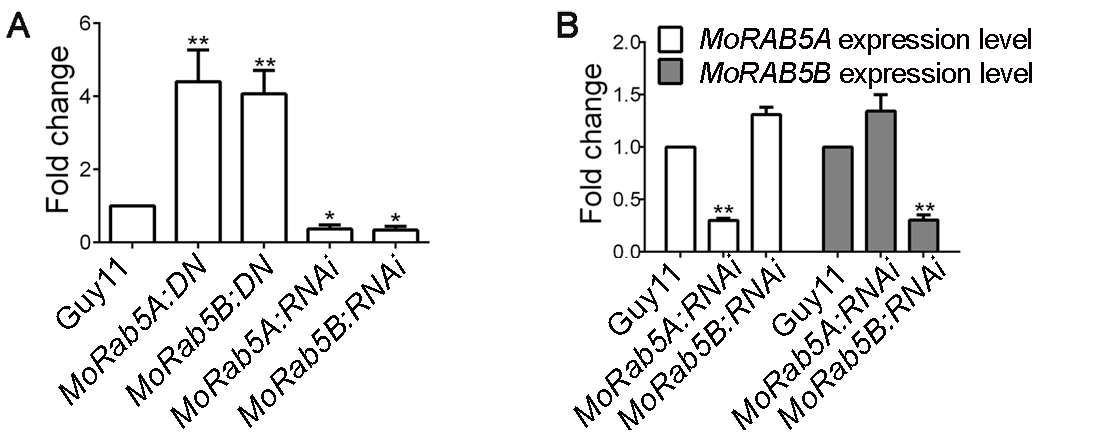
**

**Figure S1.** Identification of *MoRab5* *DN* and *RNAi* strains

**(A)** Transcription levels of *MoRAB5A and MoRAB5B* in *MoRab5A* and *MoRab5B DN* and *RNAi* strains were analyzed by qRT-PCR with β-tubulin as endogenous reference gene. **(B)** Transcription levels of *MoRAB5A and MoRAB5B* in both *MoRab5A* and *MoRab5B RNAi* strains were analyzed by qRT-PCR with β-tubulin as endogenous reference gene. At least 3 independent strains for each *DN* or *RNAi* transformant were generated and characterized, and their expression levels are essentially the same in each case. One *DN* and one *RNAi* strains are chosen as representatives to show the results in each category. The data were reproducible in three experiments and subjected to two-way ANOVA analysis comparing to the wild-type Guy11 level (^＊^= p<0.05, ^＊＊^= p<0.01).


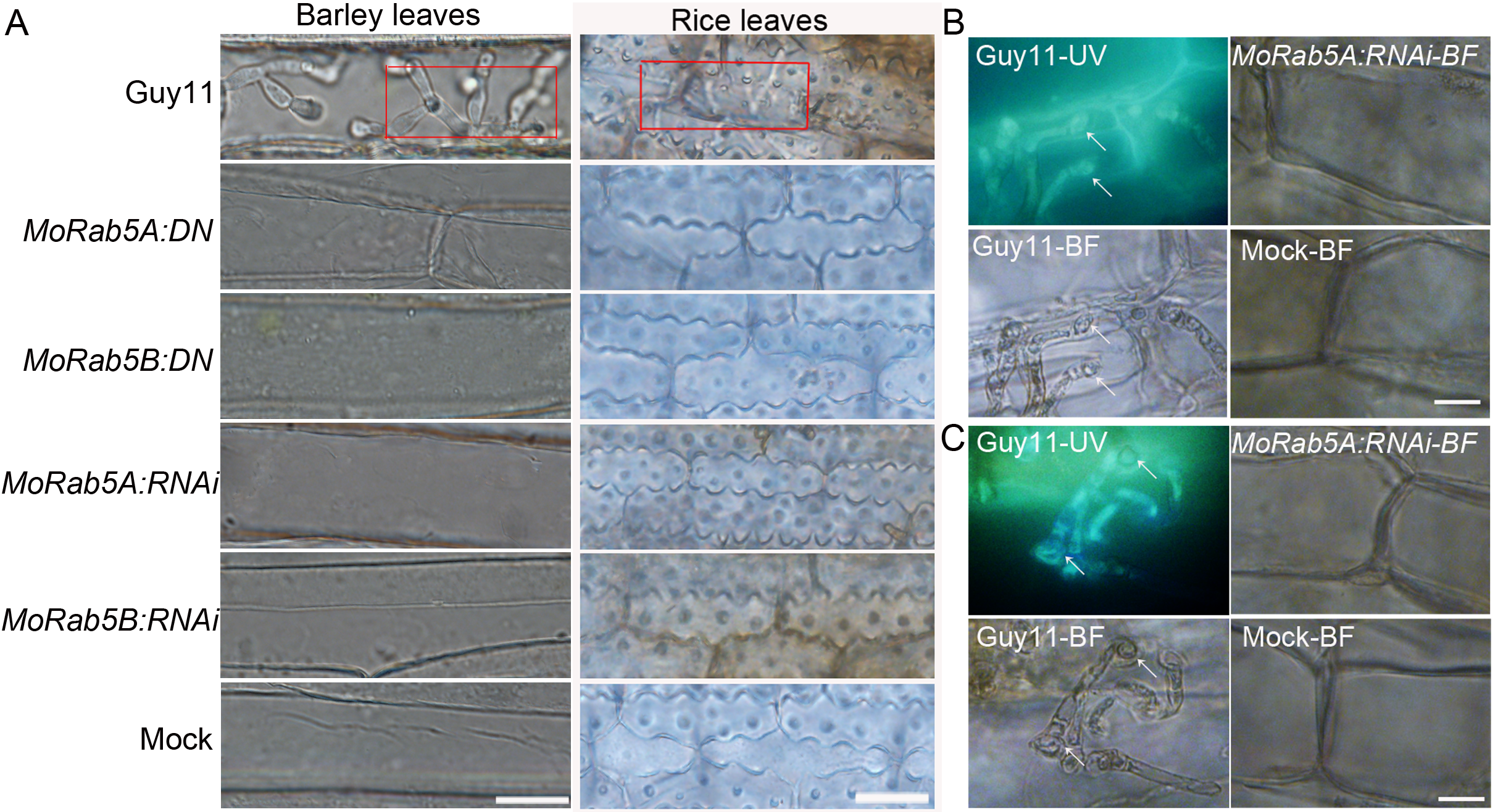


**Figure S2.** *MoRab5 DN* and *RNAi* strains are defective in producing invasive hyphae in wounded barley and rice leaves

**(A)** After 3 dpi by fresh mycelial blocks, both the epidermis cells of barley leaves and decolored superficial cells of rice leaves were collected to observe the invasive hyphal growth by bright-field microscopy. The red boxes indicate the areas in the leaves containing the invasive hyphae produced by the wild-type strain Guy11, in contrast to the leaves inoculated with the *MoRab5 DN* or *RNAi* strains where there are no visible invasive hyphae. Scale Bar = 20 μm. **(B, C)** Invasive hyphae were visualized in wounded barley **(B)** and rice leaves **(C)** by UV and bright field **(BF)** microscopy. The white arrows indicate the swollen invasive hyphae in leaves inoculated with the wild-type strain Guy11, in contrast to the *MoRab5A:RNAi* strain where there is no invasive hyphae, similar to the other *MoRab5A* and *MoRab5B DN* and *RNAi* strains that also do not produce invasive hyphae (not shown). At least 3 independent strains for each *DN* or *RNAi* transformant were generated and characterized, and their expression levels are essentially the same in each case. One *DN* and one *RNAi* strains are chosen as representatives to show the results in each category. The results were reproducible in three experiments. Scale Bar = 10 μm.

**
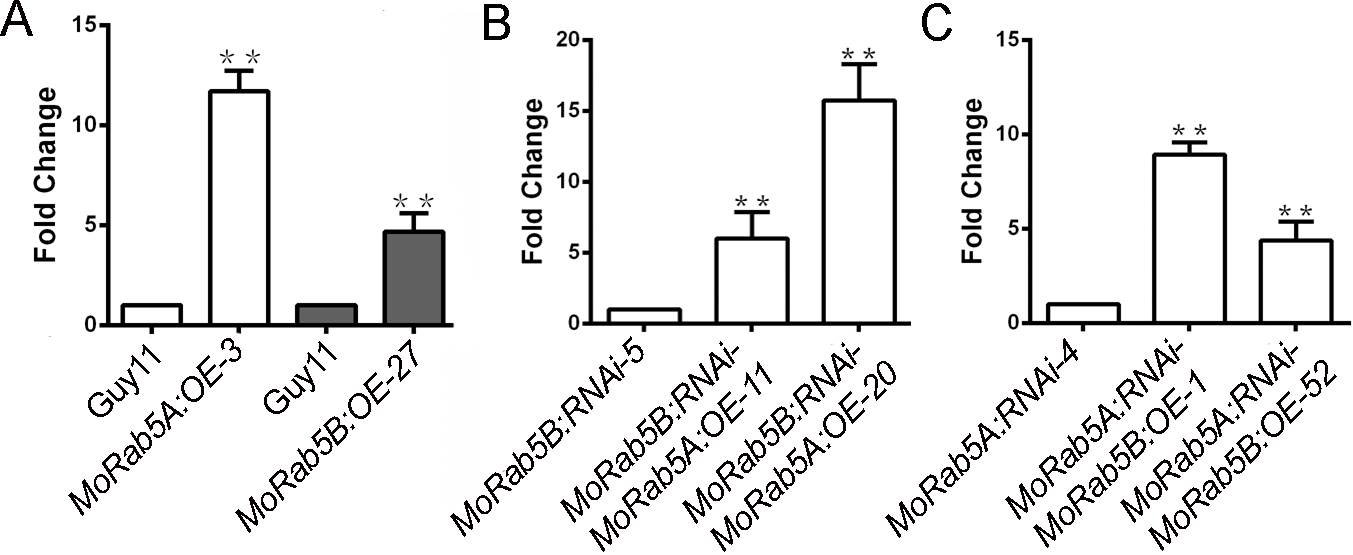
**

**Figure S3.** Transcription levels of *MoRAB5A* or *MoRAB5B* in *MoRab5B* or *MoRab5A OE* and *RNAi* strains.

**(A)** The transcription levels of *MoRAB5A* and *MoRAB5B* in Guy11, *MoRab5A:OE* and *MoRab5B:OE* strains were determined by qRT-PCR with β-tubulin as endogenous reference gene, validating *MoRAB5A* and *MoRAB5B* overexpression, respectively, in the OE strains. **(B)** Overexpression of *MoRAB5A* in two *MoRab5B:RNAi-MoRab5A:OE* strains in comparison to the control strain *MoRab5B:RNAi*, with β-tubulin as endogenous reference gene. (**C**) Overexpression of *MoRAB5B* in two *MoRab5A:RNAi-MoRab5B:OE* strains in comparison to the control strain *MoRab5A:RNAi*, with β-tubulin as endogenous reference gene. The results were reproducible in three experiments and subjected to two-way ANOVA analysis (^＊^= p<0.05, ^＊＊^= p<0.01).

**
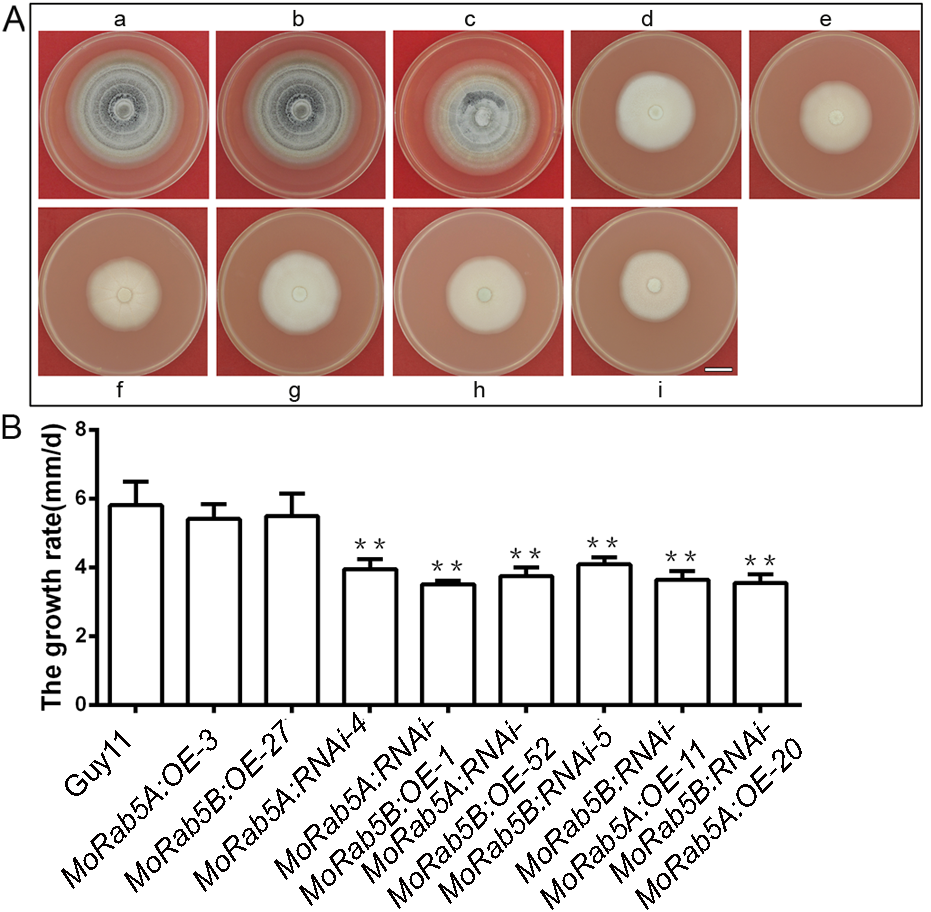
**

**Figure S4** Overexpression of *MoRAB5A* or *MoRAB5B* cannot rescue the growth defects of *MoRab5B* or *MoRab5A RNAi* strains.

**(A)** The colony morphology of *MoRab5* strains grown on CM for 10 days. a. Guy11; b. *MoRab5A:OE-3*; c. *MoRab5B:OE-27*; d. *MoRab5A:RNAi-4*;

e. *MoRab5A:RNAi-MoRab5B:OE-1*; f. *MoRab5A:RNAi-MoRab5B:OE-52*;

g. *MoRab5B:RNAi-5*; h. *MoRab5B:RNAi-MoRab5A:OE-11*;

i. *MoRab5B:RNAi-MoRab5A:OE-20*. **(B)** The average growth rates of *MoRab5* strains as indicated. The results were reproducible in three experiments and subjected to two-way ANOVA analysis comparing to the wild-type Guy11 (^＊^= p<0.05, ^＊＊^= p<0.01).


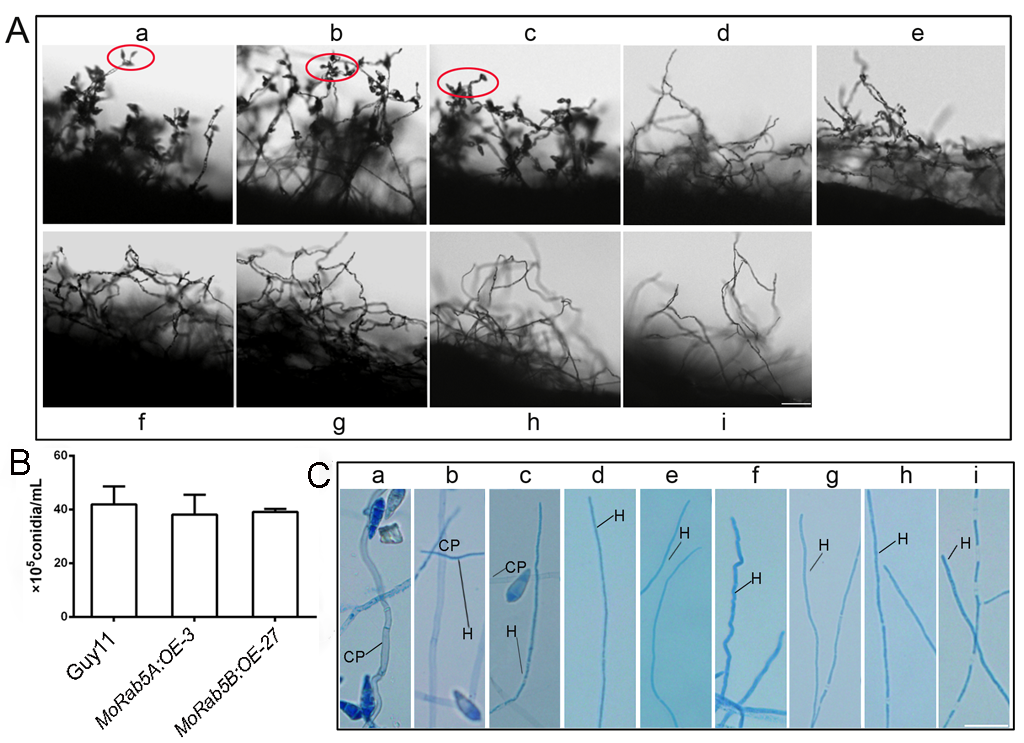


**Figure S5** Overexpression of *MoRAB5A* or *MoRAB5B* cannot rescue the conidiogenesis defects of *MoRab5B* or *MoRab5A RNAi* strains.

Visualization of conidiogenesis (A) and quantification of conidia production **(B)** by Guy11 and the *MoRab5 RNAi* and *OE* strains. a. Guy11; b. *MoRab5A:OE-3*;

c. *MoRab5B:OE-27*; d. *MoRab5A:RNAi-4*; e. *MoRab5A:RNAi-MoRab5B:OE-1*;

f. *MoRab5A:RNAi-MoRab5B:OE-52*; g. *MoRab5B:RNAi-5*;

h. *MoRab5B:RNAi-MoRab5A:OE-11*; i. *MoRab5B:RNAi-MoRab5A:OE-20*. **(C)** The same strains were stained and visualized for conidiophore structures. The results were reproducible in three experiments. CP. Conidiophore; H. hyphae. Scale bar = 20 μm.

**
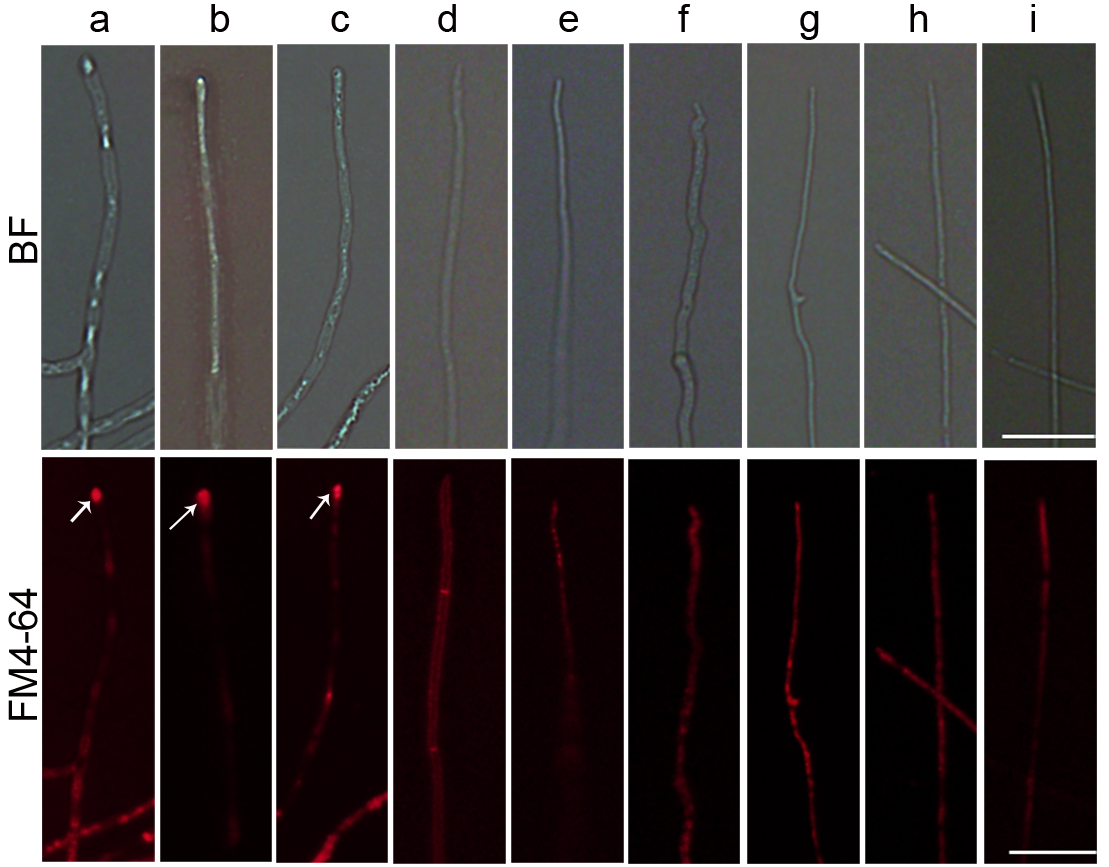
**

**Figure S6** Overexpression of *MoRAB5A* or *MoRAB5B* cannot recover the Spitzenkörper formation of *MoRab5B* or *MoRab5A RNAi* strains. The Spitzenkörper of wild-type Guy11 and the *MoRab5* *RNAi* and *OE* strains were identified by FM4-64 staining. The white arrows indicate Spitzenkörper structures. The results were reproducible in three experiments. a. Guy11; b. *MoRab5A:OE-3*; c. *MoRab5B:OE-27*;

d. *MoRab5A:RNAi-4*; e. *MoRab5A:RNAi-MoRab5B:OE-1*;

f. *MoRab5A:RNAi-MoRab5B:OE-52*; g. *MoRab5B:RNAi-5*;

h. *MoRab5B:RNAi-MoRab5A:OE-11*; i. *MoRab5B:RNAi-MoRab5A:OE-20*. Scale Bar = 20 μm.

**Table S1** main primers involved in this study

| Primers | （5’ → 3’）  Sequences | Function |
| --- | --- | --- |
| Ypt51-OF | AAAATAAGGAGCCCACCATT | *∆ypt51* haploid screening |
| Ypt51-OR | TTAGCAGCGTTCAAATCCAC |
| MoRab5AY-F | CGGGATCCATGGCCGACGGAGCCGCCA (*Bam*H) | Heterologous complementation vector construction of MoRab5AY and the transformants screening |
| MoRab5AY-R | CGGAATTCTCAACACGCGCATCCATCCT (*Eco*RⅠ) |
| MoRab5BY-F | CGGGATCCATGGCCACACGAGGACCT (*Bam*HⅠ) | Heterologous complementation vector construction of MoRab5BY and the transformants screening |
| MoRab5BY-R | CGGAATTCTTAGCACGCACAAGAACCGT (*Eco*RⅠ) |
| pYES2-F | CCCGGATCGGACTACTAG | Primers pair in pYES2 |
| pYES2-R | CTAATTACATGATGCGGC |
| *MoRab5ADN*-F1 | CGGGATCCATGGCCGACGGAGCCGCCAAAC (*Bam*HⅠ) | *MoRab5AOE* cloning |
| *MoRab5ADN*-R2 | ATAAGAATGCGGCCGCTCAACACGCGCATCCATCCT (*Not*Ⅰ) |
| *MoRab5ADN*-F1 | CGGGATCCATGGCCGACGGAGCCGCCAAAC  (*Bam*HⅠ) | *MoRab5ADN* cloning( A371T) |
| *MoRab5ADN*-R1 | GTCAAGTCGAGCTTGATGCCGACGAGCGCAAT |
| *MoRab5ADN*-F2 | ATTGCGCTCGTCGGCATCAAGCTCGACTTGAC(DN site mutation was underlined) |
| *MoRab5ADN*-R2 | ATAAGAATGCGGCCGCTCAACACGCGCATCCATCCT (*Not*Ⅰ) |
| *MoRab5BDN*-F1 | CGGGATCCATGGCCACACGAGGACCTC (*BamH*Ⅰ) | *MoRab5BOE* cloning |
| *MoRab5BDN*-R2 | ATAAGAATGCGGCCGCTTAGCACGCACAAGAACCGT (*Not*Ⅰ) |
| *MoRab5BDN*-F1 | CGGGATCCATGGCCACACGAGGACCTC (*Bam*HⅠ) | *MoRab5BDN* cloning( A398T) |
| *MoRab5BDN*-R1 | CCAAATCGAGCTTGATGCCTGCGAGCGCAAT |
| *MoRab5BDN*-F2 | ATTGCGCTCGCAGGCATCAAGCTCGATTTGG  (DN site mutation was underlined) |
| *MoRab5BDN*-R2 | ATAAGAATGCGGCCGCTTAGCACGCACAAGAACCGT (*Not*Ⅰ) |
| *MoRab5ARNAi*-F | CCGGAATTCAGGGGCTGCCTTTCTGA (*Eco*RⅠ) | *MoRab5ARNAi* cloning |
| *MoRab5ARNAi*-R | GGACTAGTCACATCCCGTGGGCCATTTA (*Spe*Ⅰ) |
| *MoRab5BRNAi*-F | GCTCTAGATGCGCAGTTTAAGTTGGT (*Xba*Ⅰ) | *MoRab5BRNAi* cloning |
| *MoRab5BRNAi*-R | ATAAGAATGCGGCCGCCCCTCGGGTGGATTTAGGCT (*Not*Ⅰ) |
| β-Tubulin-F | TCTGACTTCAGGAATGGTCGTTAC | Inference primers for qRT-PCR |
| β-Tubulin-R | AGCGGTCTGGATGTTGTTGG |
| *MoRAB5A*qRT-F | GACTCGGCGGTAGATAC | *MoRAB5A* primers for qRT-PCR |
| *MoRAB5A*qRT-R | AAACAAACCTGCTAAATGAATG |
| *MoRAB5B*qRT-F | GCTGTGCTTGATGTCAC | *MoRAB5B* primers for qRT-PCR |
| *MoRAB5B*qRT-R | TGCACGATACGAACCAT |
| MoRab5AZF | GGGTACCGGGCCCCCCCTCGAGCATCGCCAACCTCCTCCTCT (pKNT) | GFP-MoRab5A fusion vector construction |
| MoRab5AZR | TCCTCGCCCTTGCTCACCATGGCGGGAATTCACCTCGATC (GFP) |
| MoRab5AGOF | CACTCACGGCATGGACGAGCTGTACAAGATGGCCGACGGAGCCGCCA (GFP) |
| MoRab5AGOR | CCCCCGGGCTGCAGGAATTCTCGCCATTTCCACGGGTC (pKNT) |
| MoRab5BZF | GGGTACCGGGCCCCCCCTCGAGCATCTCCTTGCCACCACC (pKNT) | GFP-MoRab5B fusion vector construction |
| MoRab5BZR | TCCTCGCCCTTGCTCACCATGTTGATTGTGTACGAAAATA (GFP) |
| MoRab5BGOF | CACTCACGGCATGGACGAGCTGTACAAGATGGCCACACGAGGACCTCC (GFP) |
| MoRab5BGOR | CCCCCGGGCTGCAGGAATTCCCAGACAGGAGCAGAACAA (pKNT) |
